# Case of Diffused Spurious Aneurism

**Published:** 1831-01-01

**Authors:** 


### LXIX.

### GUY'S HOSPITAL.

#### DIFFUSED SPURIOUS ANEURISM.

Oct. 21st, 1830, Thomas Noon, æt. 31, was admitted under Mr. Morgan, about eight weeks ago, for an aneurismal tumour of the left arm, the consequence of a punctured artery during bleeding, which had been done in the country a fortnight before. The limb was then greatly inflamed and swollen in the neighbourhood of the elbow, the tumour extending upwards, chiefly in front, as far as the tendons of the pectoralis major and latissimus dorsi, and bounded below by the fascia of the biceps. At the time he was bled, the patient states it was immediately evident that an artery had been wounded, by the great impetuosity of the current of blood, which was thrown out in jets to a considerable distance. By the application of firm pressure over the wound, the flow of blood was readily stopped. It, however, produced very considerable pain, and swelling instantly took place and spread up the arm. Leeches, in great numbers, were required to reduce the inflammation, and, since his admission, he has undergone general antiphlogistic treatment, by purgatives and saline remedies.

The present state of the disease is as follows: There is a large firm tumour diffused over the anterior surface of the upper arm, from an inch to two inches below the elbow-joint, upwards to the pectoral insertion. At the lower and inner part of this tumour, just above the internal condyle, there is less hardness than in the rest of its extent, and here, alone, pretty forcible arterial pulsation is distinctly felt by the finger, and a whizzing noise is plainly communicated to the ear, either by mediate or immediate auscultation. The swelling extends some distance outwards around the arm, but gradually disappears posteriorly. No distinct or circumscribed tumour exists in the front of the joint, nor any communication of the artery with the vein which was selected for the operation, the puncture of which healed perfectly in the usual time. The motions of the elbow-joint are of course completely suspended, and the fore-arm is kept slightly bent upon the upper. Pain is very trifling, but the patient constantly feels numbness and tingling, which often at periods amount to such, along the outer side of the fore-arm, and to the extremities of those fingers supplied by the median nerve, viz. on both sides of the thumb and two external fingers, and on the external side of the ring finger, whilst the inner side of this and both sides of the little finger, (receiving branches from the ulnar nerve) are quite free from either and possess perfect sensation, which is somewhat blunted in the former; for friction readily felt on the internal side of the ring finger, is with difficulty distinguished on its outer side. The dimensions of the swelling have scarcely enlarged latterly; the patient however, thinks, they are rather less than they have been. His health has not been much affected, and it is now good, and he has been for some time desirous that any operation, though necessary, should be performed. Thi was done by Mr. Morgan in the following method. A tourniquet being firmly applied around the arm, an incision was made, with a double-edged scalpel, in front of the elbow-joint, about four inches in length, in order to expose the artery where it was wounded. An immense quantity of coagula, black, and having no appearance of organization, was fouud effused beneath the cellular tissue, by which the tumour was formed, and which was removed by the fingers. A *transverse* wound of the brachial artery was discovered, just above the division of the vessel, the edges of which were some distance apart, and had undergone slight ulceration. Two ligatures were applied on the artery, one above and the other below this opening, without much difficulty, as the trunk was not farther from the surface than ordinarily, when the coagulated blood was cleared away. The external wound was brought together by three sutures and strips of plaister, applied at a little distance from each other, and a light bread poultice was to be put on when the man should be in bed. A copious flow of venous blood followed the first incision, and the hand, which had previously been livid, became, at the close of the operation, remarkably blanched. The patient was disposed to faint at one time, but bore the whole well and most manfully.

Three hours after the operation the man was comfortable, and had recovered from its immediate effects. The numbness of the fingers had already ceased; his skin was cool, and pulse not more than 70.

Oct. 22d. Pain in left side of chest increased, in some measure, on inspiration, which the patient himself attributes to his being in the recumbent position, and inclining rather to that side. His countenance is somewhat distressed, and he got no sleep in the night; the limb, however, has been easy, and entirely free from tingling or numbness, and is so now, excepting some smarting of the wound; hand and fingers feel hot to the patient—they are of natural temperature, the whole fore-arm being enveloped in flannels, &c. The pulse is distinguishable at the wrist, and is synchronous with the opposite radial, though of course very feeble—skin warm, and perspiring freely—pulse full, but soft, about 80—tongue rather yellowish, furred, and dry—has made water freely, but the bowels have not acted. Mr. Morgan ordered an aperient mixture, some of which was to be taken immediately, and repeated if necessary. Cat. lini to the arm.

23d. The bowels were opened this morning early, after four doses of the medicine. He slept none last night, and has a continuance of pain in chest—no cough or expectoration. In other respects he is doing well, and is quite easy—no swelling or redness about the elbow.

24th. The pain of chest has left him—he got six hours continued sleep in the night, and, in fact, is so much better, that we found him in the afternoon reading a book—limb in no pain, and feels of proper warmth—pulse 80—skin moist and temperate—bowels open. Straps were removed from wound yesterday, which looked well, although there was no union by the adhesive process.

25th. Doing well.

28th. Has continued well in every particular, and to-day took a mutton chop for dinner. The radial pulse on the sound side is firm and tolerably full—that on the opposite cannot now be distinguished, but was easily felt two days ago by Mr. Morgan, and, according to the patient's account, repeatedly by the dresser subsequently.

29th. Pulse at left wrist felt to-day feeble, but regular, and quite simultaneous with the heart's action.

31st. One ligature to-day came away.

Nov. 6th. Patient continues remarkably well. The pulse of the radial artery has gradually become more and more indistinct, and is now to be felt only at times, and then it is found to be excessively feeble—the remaining ligature has separated, and the wound, which has been hitherto poulticed, is ordered to be strapped. The wound has not contracted in size, but looks healthy. The tumefaction and hardness have subsided on the upper arm, which is now reduced to the same dimensions, and is equally soft with the other.

11th. He has sat up daily since our last account—is quite well in health, and very much gratified at the favourable result of his case. There is no enlargement left in the upper arm; the motions of the wrist and fingers are returned, and those of the elbow too in a limited degree; but there is every prospect that, in course of time, the latter joint will be rendered as useful as before, by exercising judiciously the natural motions of the limb.

17th. This day he was discharged from the hospital well.

The man has since appeared at the hospital, and the utility of the arm is nearly restored, some stiffness only of the elbow being perceptible.

Beta.

